# Effects of *Spirogyra jaoensis* as a dietary supplement on growth, pectoralis muscle performance, and small intestine morphology of broiler chickens

**DOI:** 10.14202/vetworld.2019.1233-1239

**Published:** 2019-08-11

**Authors:** H. T. Saragih, A. A. K. Muhamad, Alfianto Alfianto, F. Viniwidihastuti, L. F. Untari, I. Lesmana, H. Widyatmoko, Z. Rohmah

**Affiliations:** 1 Department of Animal Development Structure, Faculty of Biology, Universitas Gadjah Mada, Yogyakarta, 55281, Indonesia; 2 Department of Research and Development, Sari Rosa Asih Company, Yogyakarta, Indonesia

**Keywords:** broiler chicken, feed supplement, pectoralis muscle growth, small intestine, *Spirogyra jaoensis*

## Abstract

**Aim:**

This study aimed to examine the effect of dietary *Spirogyra jaoensis* in starter feed on growth performance, pectoralis muscle (PM) growth, and small intestine morphology of broiler chickens.

**Materials and Methods:**

One hundred twenty one-day-old Cobb-500 broilers (body weight 46±2.6 g) were divided into four equal groups with 3 replicates in each group and given basal feed supplemented with dried *S. jaoensis* at doses of 0%, 0.5%, 1%, or 2%. The treatment was carried out until the chickens were 18 days old to examine growth of broiler chicks at starter period (8-21 days old).

**Results:**

Supplementation with *S. jaoensis* at doses of 0.5% and 1% resulted in increased weight and improved feed conversion ratio compared to the control group. At the end of treatment, chickens fed with 0.5% and 1% *S. jaoensis* weighed 428.3±47.8 g and 426.9±31.8 g, respectively, and were significantly heavier than the control group (373.1±44.1 g). Furthermore, parameters related to PM growth and small intestine morphology of chickens supplemented with 0.5% *S. jaoensis* in basal feed were improved compared to the control group.

**Conclusion:**

The results of this research indicate that *S. jaoensis* at a dose of 0.5% improves growth performance, PM growth, and small intestine morphology in broiler chickens.

## Introduction

In Indonesia, market demand for protein derived from animals is very high. The four main sources of animal-derived protein are pigs, cows, goats, and chickens, the biggest being chicken (*Gallus gallus domesticus* L.) with a consumption rate of 4.01 kg/person/year or 85% of national fresh meat consumption per capita [1]. To satisfy this high market demand, the poultry industry needs to enhance its production. The yield of production reflects the growth rate and weight gain (WG) of chickens, especially the pectoralis muscle (PM), which represents the majority of consumed muscle [2,3]. In an effort to maximize the performance of chickens to meet the demands of consumers, nutritional factors play a pivotal part along with genetic factors [4-8]. A previous study reported that high nutritive feed is important for health and development of the digestive system of chickens, which, in turn, will promote weight growth, especially if given immediately after hatching [9].

The important characteristics of chicken feed for optimum growth are the availability and ratio of protein, carbohydrates, and fats [10]. The need for protein in chicken feed is satisfied by soybean meal and corn ingredients. At present, soybean meal ingredients used by chicken feed manufacturers in Indonesia are almost completely obtained through importation [11]. Hence, alternative material is needed to forestall any difficulties with the availability of soybean meal in the future.

*Spirogyra* is a genus of filamentous algae, which normally live floating freely in freshwater. The chemical composition of *Spirogyra* spp. consists of 12-24% protein, 43-62% carbohydrate, and 15-21% fat [12]. In addition, *Spirogyra neglecta* is known to have antibacterial, anticancer, antidiabetic, and antioxidant properties in the rat model [13-16]. *S. neglecta* also has immunomodulatory activity when tested on animal cell cultures [17]. A species of *Spirogyra* that is commonly found in Java, Indonesia, is *Spirogyra jaoensis*, although this alga has not been widely studied either for health or as a dietary component for chickens.

This study aimed, to explore the potential of *S. jaoensis* as a new feed supplement and to determine the effect of dietary *S. jaoensis* on growth performance, PM growth, and small intestine morphology of broiler chickens.

## Materials and Methods

### Ethical approval

Ethical approval was obtained from the Universitas Gadjah Mada Integrated Research and Testing Laboratory (LPPT UGM), with No. 00039/04/LPPT/IV/2018.

### Treatment feed formulation and preparation

*Spirogyra* spp. algae were obtained from fields in Sleman Regency, Yogyakarta, Indonesia, and identified as *S. jaoensis* in the Plant Systematics Laboratory, Faculty of Biology, Gadjah Mada University. Proximate composition of *S. jaoensis* was determined before feed treatment (Table-1). Ash, moisture, and carbohydrate content were determined with methods according to the Association of Official Agricultural Chemists [18]. Crude protein, fat, and fiber contents were analyzed with methods described by Pearson [19]. *S. jaoensis* was washed and air-dried at room temperature for 1 day and then dried in incubator at 50°C for 3 days. The dried *S. jaoensis* was then pulverized to a fine powder, using a domestic blender, which was used to supplement the basal feed. A bomb calorimeter test was also conducted to calculate the metabolizable energy of *S. jaoensis* supplemented feed (Table-1).

**Table 1:** Nutritional content of *Spirogyra jaoensis.*

Parameter	Value
Crude protein (%)	18
Crude fiber (%)	37
Crude fat (%)	5.5
Ash (%)	28
Moisture (%)	11
Metabolizable energy (kcal/kg)	2174

### Feed treatment

One hundred twenty 1-day-old chicks (DOCs) with an average weight of 46±2.6 g were obtained from Sabar Poultry Shop, Indonesia. Before treatment, the DOCs were allotted randomly into four groups with 3 replicates in each group and a sex ratio of 50:50 within groups. The cages were of a size suitable to house 8-12 DOCs/m^2^. The cages were kept in a temperature-controlled room. The temperature was maintained at 29-31°C and checked twice a day. Cages and room conditions were compiled rearing standards for broiler DOCs. Feed and water were given *ad libitum*, and fresh feed and water were supplied in the morning and evening. DOCs were acclimatized until 3 days old. To examine the effect of *S. jaoensis* supplementation on growth of broiler chicks at starter period (8-21 days old), the treatment feed was given to DOCs until 18 days old. Group 1 was designated as the K0 group and given basal feed without any supplementation. Groups 2 (P1), 3 (P2), and 4 (P3) were given basal feed supplemented with 0.5%, 1%, and 2% *S. jaoensis*, respectively.

All the chicks were weighed at age 0 (post-hatch), 3, 6 9, 12, 15, and 18 days old. The weights of feed provided and left uneaten were recorded daily to calculate the amount of feed consumed (feed intake [FI]). The feed conversion ratio (FCR) was calculated as the FI needed to gain 1 kg in weight (WG) [20,21]:

FCR = FI/WG

### Measurement of small intestine morphology

On the final day of treatment (day 18), all chicks were fasted and eight from each group were euthanized. The small intestine (duodenum, jejunum, and ileum) was excised and prepared for histological examination using the paraffin method [22]. Histological slides were visualized by periodic acid–Schiff staining [23]. Small intestine microscopic morphology was documented using a Leica microscope digital camera system and software. Villus height, crypt depth, and goblet cell area were measured using Image Raster 3.0 software (Miconos Transdata, Indonesia) [24].

### PM observation

The PM was excised, and the left half of the muscle was weighed while the right half was photographed and measured for PM area with ImageJ software (NIH-USA). For histological analysis, 3 cm×3 cm of muscle tissue was processed using the paraffin method and hematoxylin-eosin staining. The histological slides of muscle were then observed and measured for fasciculus and myofiber size/area using ImageJ [25].

### Statistical analysis

Data on body weight, FCR, small intestine morphology, and PM parameters were analyzed using one-way analysis of variance (ANOVA), followed by Tukey’s test at a confidence threshold of 5%.

## Results

### Growth performance

**Figure-1:**
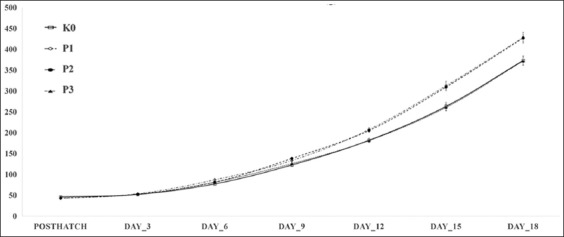
Effects of dietary *Spirogyra jaoensis* on bodyweight (± standard error median) of broiler chickens from post-hatch until 18 days of age. K0=Control group with basal feed, P1=Chicks treated with basal feed+*S. jaoensis* (0.5% of feed), P2=Chicks treated with basal feed+*S. jaoensis* (1% of feed), P3=Chicks treated with basal feed+*S. jaoensis* (2% of feed).

The effects of various doses of *S. jaoensis* on growth performance of broiler chickens are shown in Figure-1 and Table-2. Chickens fed basal feed supplemented with *S. jaoensis* at 0.5% and 1% (P1 and P2) had higher WG compared to both the K0 group and the treatment group supplemented with 2% (P3) *S. jaoensis*. The increased WG with 0.5% and 1% *S. jaoensis* supplementation compared to the K0 group began at the age of 6 days old and continued to 18 days old. WG of broilers fed with *S. jaoensis* at doses of 0.5%, 1%, and 2% was 29.7±18 g/day, 25.5±11.8 g/day, and 23.7±16.6 g/day, respectively. These rates are greater than that of the K0 group (19.3±10.5 g/day), although none of these differences are significant. The FCR values of all four groups were similar.

**Table 2:** Effects of dietary *Spirogyra jaoensis* on the performance of broiler chickens at 18 days old.

Variable	K0	P1	P2	P3
Feed intake (g/day)	28.81±12.5^ns^	35.79±14.08^ns^	35.40±13.31^ns^	30.16±13.6^ns^
Weight gain (g/day)	19.28±10.5^ns^	29.56±17.98^ns^	25.52±11.79^ns^	23.69±16.55^ns^
FCR (mm^2^)	1.76±0.95^ns^	1.45±0.75^ns^	1.51±0.54^ns^	1.44±0.56^ns^

Numbers in a particular row that is followed by different letters are significantly different from each other (p<0.05). ns=no significant differences within the row. FCR=Feed conversion ratio

### PM growth

**Figure-2:**
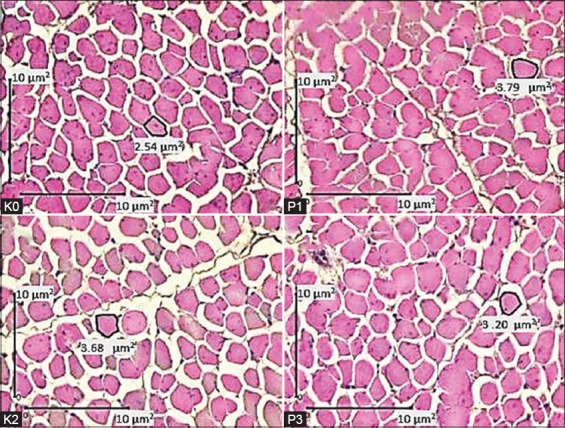
Sections of pectoralis muscle tissue of broiler chickens. K0=Control group with basal feed, P1=Chicks treated with basal feed+*Spirogyra jaoensis* (0.5% of feed), P2=Chicks treated with basal feed+*S. jaoensis* (1% of feed), P3=Chicks treated with basal feed+*S. jaoensis* (2% of feed). In the control, the myofiber area is smaller than that in chicks fed with *S. jaoensis*. Hematoxylin and eosin staining.

The effects of various doses of *S. jaoensis* feed supplementation on PM growth of broiler chickens are presented in Table-3 and Figure-2. The weight, area, fasciculus, and myofiber area of PM of the P1 and P2 treatment groups were higher than those of the K0 and P3 treatment groups. The muscle weight of P1 (35.5±3.6 g) was significantly higher than those of K0 (28.04±2.8 g), P2 (27.7±1.7 g), and P3 (28.8±3.6 g). The PM area of treatment groups was 61.6±8.8 mm^2^, 53.6±8.4 mm^2^, and 53.9±7.8 mm^2^ for P1, P2, and P3, respectively, and 48.4±4.2 mm^2^ for K0. The PM area values of P1, P2, and P3 were all significantly higher than that of K0 (p<0.05).

**Table 3:** Effects of dietary *Spirogyra jaoensis* on the pectoralis muscle of broiler chickens at 18 days old.

Variable	K0	P1	P2	P3	p-value
Muscle weight (g)	28.04±2.82^a^	35.51±3.61^b^	27.73±1.71^a^	28.76±3.59^a^	0.001
Muscle area (mm^2^)	48.37±4.24^a^	61.62±8.76^b^	53.58±8.44^ab^	53.88±7.80^ab^	0.039
Fasciculus area (mm^2^)	536.75±45.88^a^	683.25±19.31^b^	579.91±28.64^a^	563.96±26.54^a^	0.000
Myofiber area (mm^2^)	3.47±0.27^a^	4.38±0.14^b^	3.38±0.34^a^	3.49±0.19^a^	0.000

Numbers in a particular row that is followed by different letters are significantly different from each other (p<0.05)

### Small intestine morphology

The effect of *S. jaoensis* in basal feed on intestinal morphology of broiler chickens is shown in Table-4. The villus of the duodenum in P1 (733.0±41.1 µm) was significantly higher than those of K0 (663.4±39.1 µm), P2 (671.6±13.9 µm), and P3 (656.0±29.7 µm); this was also the case for the villus of the jejunum. Moreover, the villus of ileum in P1 and P2 was significantly higher than those in K0 and P3. Crypt depth in the duodenum and ileum of treatment groups was higher than that of control, although there was no significant difference in jejunum crypt depth between any groups. In general, the number and size of goblet cells in the small intestine of treatment groups were higher than those of control, especially in P1 and P2.

**Table 4:** Effects of dietary *Spirogyra jaoensis* on villus height, crypt depth, villus/crypt ratio, number of goblet cells, and area of goblet cells of broiler chickens at 18 days old.

Variable	K0	P1	P2	P3	P-value
Duodenum					
Villus height (μm)	663.4±39.09^a^	733±41.13^b^	671.6±13.87^a^	656±29.64^a^	0.019
Crypt depth (μm)	104.2±11.03^a^	136.8±29.75^b^	118.2±13.99^ab^	106±7.78^ab^	0.046
Ratio V/C	6.37±0.49^a^	5.48±0.80^a^	5.74±0.72^a^	6.16±0.27^a^	0.132
Number of goblet cells/100 cells	53.6±3.05^a^	67.6±1.82^c^	59.2±1.92^ab^	55.8±2.28^ab^	0.000
Area of goblet cells (μm^2^)	7.08±0.26^a^	7.88±0.31^b^	7.09±0.22^a^	7.04±0.15^a^	0.001
Jejunum					
Villus height (μm)	466.59±10.53^a^	550.76±37.16^b^	488.15±5.71^a^	473.95±3.52^a^	0.000
Crypt depth (μm)	105.33±3.97^a^	105.54±7.22^a^	98.26±2.26^a^	98.87±2.20^a^	0.092
Ratio V/C	4.43±0.14^a^	5.22±0.20^c^	4.97±0.17^bc^	4.79±0.07^b^	0.000
Number of goblet cells/100 cells	26.6±1.14^ab^	30.2±1.48^c^	27.2±0.84^ab^	24.8±0.84^a^	0.001
Area of goblet cells (μm^2^)	6.38±0.10^a^	7.78±0.11^c^	7.07±0.16^b^	6.54±0.04^a^	0.000
Ileum					
Villus height (μm)	384.19±6.77^a^	420.96±10.05^c^	399.13±7.13^b^	377.23±5.55^a^	0.000
Crypt depth (μm)	82.15±2.58^ab^	84.93±3.37^bc^	86.93±1.65^c^	79.47±1.52^a^	0.029
Ratio V/C	4.68±0.09^a^	4.96±0.11^b^	4.59±0.06^a^	4.75±0.09^a^	0.001
Number of goblet cells/100 cells	30.20±2.28^a^	38.8±1.09^c^	35.40±1.14^b^	30.8±1.64^a^	0.000
Area of goblet cells (μm^2^)	6.89±0.12^a^	8.91±0.25^c^	7.59±0.09^b^	6.99±0.11^a^	0.000

Numbers in a particular row that is followed by different letters are significantly different from each other (p<0.05). V/C=Villus height/crypt depth

## Discussion

Algae have many benefits as food sources and animal feed additives [26-29]. In chickens, algae are known to increase immunity, help the digestive system, and have a positive effect on the quality of meat products and chicken eggs [30-32].

The previous studies reported that the protein content in chicken feed had a considerable influence on the growth of chickens and also increased the efficiency of the digestive system [33,34]. In our study, supplementation with *S. jaoensis* in broiler chickens could enhance weight increase and improve FCR compared to the control group. These results were consistent with previous research reporting that the administration of *S. ellipsospora* could also stimulate WG in broiler chickens [35]. *S. jaoensis* used in this research has a content of 16% protein, 36% carbohydrate, and 11% lipid (Table-1), which we believe influenced the growth of broiler chickens.

**Figure-3:**
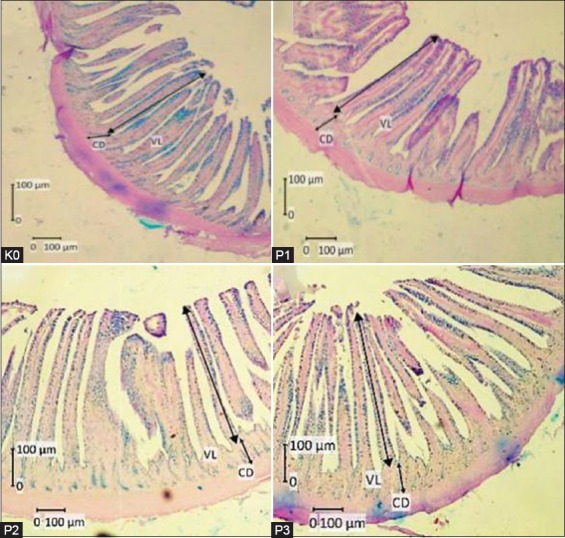
Sections of jejunum tissue of broiler chickens. K0=Control group with basal feed, P1=Chicks treated with basal feed+*Spirogyra jaoensis* (0.5% of feed), P2=Chicks treated with basal feed+*S. jaoensis* (1% of feed), P3=Chicks treated with basal feed+*S. jaoensis* (2% of feed). In the control, the villi are shorter and thicker than those in chicks fed with *S. jaoensis*, CD=Crypt depth, VL=Villus length, Periodic acid–Schiff staining.

PM growth is an important indicator of the success of broiler chicken growth [36]. The PM is often used as a parameter for chicken muscle growth due to its lack of fat accumulation [37]. A good level of protein in chicken feed is very important for PM growth: Proteins are known to stimulate the proliferation of satellite cells to regenerate myofiber cells for better muscle growth [38]. In this research, we observed that muscle growth of pectoralis broiler chickens supplemented with *S. jaoensis* in feed was improved (Table-3 and Figure-3). Even though there were no significant differences for FCR, daily growth, or FI, there was a tendency for body and PM weight to increase in treatment groups. This can be attributed to the high protein, lipid, and carbohydrate content of *S. jaoensis*. These components increased the calorie count in basal feed (Table-5), which, thus, became an improved source of energy for growth.

**Table 5:** Basal feed formulation, treatment feed proximate composition, and calorimetric analysis for feed treatment with *Spirogyra jaoensis* supplementation.

Composition of feed (%)	Starter			

	*Spirogyra jaoensis* (%)			

	0	0.5	1	2
Corn	49.0	49.0	49.0	49.0
Soybean meal	29.0	29	29	29
Rice bran	9.8	9.8	9.8	9.8
Full-fat soya	5.4	5.4	5.4	5.4
Crude palm oil	3.0	3.0	3.0	3.0
Dicalcium phosphate	2.37	2.37	2.37	2.37
Premix vitamin	0.03	0.03	0.03	0.03
Premix mineral	0.06	0.06	0.06	0.06
D,L-methionine	0.22	0.22	0.22	0.22
NaCl	0.32	0.32	0.32	0.32
Calcite	0.5	0.5	0.5	0.5
L-lysine HCl	0.1	0.1	0.1	0.1
L-threonine	0.04	0.04	0.04	0.04
Choline chloride 60%	0.16	0.16	0.16	0.16
Calculated composition				
Metabolizable energy of poultry (kcal/kg)	2904.02	2914.89	2925.76	2947.5
Crude protein (%)	20.23	20.23	20.23	20.23
Crude fat (%)	8.30	8.30	8.30	8.30
Fiber (%)	3.37	3.37	3.37	3.37
Lysine (%)	1.22	1.22	1.22	1.22
Methionine (%)	0.53	0.53	0.53	0.53
Methionine+cysteine (%)	0.86	0.86	0.86	0.86
Calcium (%)	1	1	1	1
Phosphorus, total (%)	0.95	0.95	0.95	0.95
Phosphorus, available (%)	0.5	0.5	0.5	0.5
Sodium (%)	0.15	0.15	0.15	0.15
Chloride (%)	0.23	0.23	0.23	0.23

The small intestine is an important site for the absorption process in chickens. It consists of the duodenum, jejunum, and ileum, all of which have absorption functions. Efficiency in the absorption of food nutrients is influenced by tissue structure in the small intestine, especially in villus length, crypt depth, and optimal goblet cell numbers for secreting mucin. Villi in the small intestine play a role in the absorption of nutrients by increasing the surface area of the intestine [39]. The role of the crypt is to accelerate the repair of villus tissue that has been damaged by exfoliation, inflammation, or toxins originating from pathogens. Villus height is directly proportional to crypt depth and can be used as an indicator of good small intestinal morphology: A low villus height/crypt depth ratio indicates good absorption of nutrients [21]. In this research, the morphology of the small intestine in treatment groups (Table-4 and Figure-1) showed an improvement over the control group. This suggested that fiber present in *S. jaoensis* had a positive impact on intestinal morphology, which, in turn, made the small intestine more effective in nutrient absorption. This result is in agreement with a previous report that the presence of high free fiber from *Trichoderma* fermented wheat bran in diet could improve small intestine morphology in broiler chickens [40].

Goblet cells also have an important role in synthesizing mucin, a glycoprotein that helps to maintain the ecology of the intestine by excluding germs and preventing epithelial degradation [41]. The wider the area of goblet cells and the greater the number of goblet cells, the more effective is the protection of the surface area of villi from pathogen attack. The results of our research showed that administration of *S. jaoensis* increased the number and area of goblet cells (Table-4), suggesting that mucin production in the small intestine is higher in the treatment groups. Elevated intestinal mucin along with higher epithelial turnover may increase the effectiveness of the small intestine in nutrient absorption [42].

## Conclusion

This research indicates that supplementing feed with the alga *S. jaoensis* at a dose of 0.5% has the potential to improve growth performance, PM growth, and small intestine morphology in broiler chickens.

## Authors’ Contributions

HTS, AAKM, AA, and FV contributed equally to the experimentation. HTS, IL, and ZR wrote and edited the article. HTS, LFU, and HW equally designed the experiment. All authors read and approved the final manuscript.
